# The impact of comorbidity on overall survival in elderly nasopharyngeal carcinoma patients: a National Cancer Data Base analysis

**DOI:** 10.1002/cam4.1377

**Published:** 2018-03-01

**Authors:** Ying Huang, Wei Chen, Waqar Haque, Vivek Verma, Yan Xing, Bin S. Teh, Edward Brian Butler

**Affiliations:** ^1^ State Key Laboratory of Oncology in South China Department of Radiation Oncology Collaborative Innovation Center for Cancer Medicine Sun Yat‐sen University Cancer Center Guangzhou China; ^2^ Department of Radiation Oncology Houston Methodist Hospital Houston Texas USA; ^3^ Department of General Surgery Guangdong General Hospital Guangzhou China; ^4^ Department of Radiation Oncology Allegheny General Hospital Pittsburgh Pennsylvania USA; ^5^ Department of Hematology & Oncology Houston Methodist Hospital Houston TX USA

**Keywords:** Comorbidity, elderly, nasopharyngeal carcinoma, National Cancer Data Base (NCDB), prognosis

## Abstract

The number of elderly patients with cancer is increasing. Medical comorbidities are more common in this population. Little is known regarding the prognostic relevance of comorbidities in elderly patients with nasopharyngeal carcinoma (NPC). Using the National Cancer Data Base (NCDB), we queried patients age >65 years diagnosed with NPC and treated with definitive radiation between 2004 and 2012 to examine the association between comorbidity and survival outcomes. Comorbidity was assessed with the Charlson Comorbidity Index (CCI). The influence of comorbidity on overall survival (OS) was evaluated. Cox proportional hazards model was used to study the impact of comorbidity on OS. A total of 1137 patients met the specified criteria. Median follow‐up was 61.2 months. Five‐year OS was 50.4%. Comorbidities were present in 22.4% of patients, with 17.6% of patients having a CCI score of 1% and 4.8% having a CCI score of ≥2. Patients with a CCI score of 0 had significantly higher 5‐year OS than patients with a CCI score of 1 or ≥2 (53.1% vs. 42.2% vs. 32.9%, *P* < 0.001). In multivariate analysis, CCI was a statistically significant independent prognostic factor for the risk of death of all causes for patients with a CCI score of 1 (hazard ratio [HR]: 1.242; 95% confidence interval [CI]: 1.002–1.539) or CCI score of ≥2 (HR: 1.625; 95% CI: 1.157–2.283) when compared to patients with a CCI score of 0. Comorbidity as measured by CCI is a strong independent prognostic factor for OS in elderly patients with NPC and lends support to the inclusion of comorbidity assessment due to its prognostic value when treating elderly patients with NPC.

## Introduction

Nasopharyngeal carcinoma (NPC) differs from other head and neck cancers because of its distinct epidemiology and geographic distribution 1. Radiation therapy (RT) with or without chemotherapy has become standard of care treatment for NPC 2. As the world's population continues to age, a greater proportion of patients with cancer are elderly 3. However, only a small proportion of elderly patients can be enrolled in clinical trials because of strict inclusion and exclusion criteria, making the optimal management of elderly patients with cancer unclear. As tolerance to high‐dose RT or combined radio‐chemotherapy program may be more challenging for elderly patients, who often may have reduced organ functions and multiple comorbidities, a reduction in treatment intensity for these patients may be beneficial, especially in patients with a poor performance status 4, 5.

Although the most prevalent risk factors for the development of head and neck cancer are the use of tobacco and alcohol, these substances are also associated with other significant systemic comorbidities such as cardiovascular, pulmonary, gastrointestinal, and metabolic diseases, each of which may adversely impact treatment tolerance and influence prognosis. Furthermore, elderly patients are a heterogeneous population in regard to their performance status. As the population ages, the importance of these standard prognostic factors will be increasingly important and must be reassessed. Medical comorbidities have been associated with the care of the patient, selection of initial treatment, the incidence of complications following radical treatment, and evaluation of treatment effectiveness 6, 7, 8, 9. The patient's underlying comorbidities have been also demonstrated to affect the survival of patients with head and neck cancer, independent of stage 10, 11, 12, 13. Although studies have demonstrated that there is an independent link between outcome and comorbidity status for patients with NPC, this has primarily been shown by Asian studies 14, 15, 16, 17, while a single small American study failed to show any link between comorbidity and outcome for NPC patients 18. As the histology of NPC frequently seen in Asia is typically nonkeratinizing carcinoma, as opposed to the keratinizing squamous cell carcinoma subtype more commonly seen in the United States, it is possible that comorbidity may not have as much of an impact on outcome for the more aggressive subtype that is frequently seen in the United States 19.

To our knowledge, no studies have yet investigated the impact of comorbidity on survival in a large cohort of NPC patients within the United States, especially in elderly patients. The aims of this study were to evaluate the presence of comorbid conditions for elderly patients with NPC receiving definitive RT within the United States and to investigate the where these patient's medical comorbidities independently predicted for overall survival (OS).

## Materials and Methods

### Study population

This study was a retrospective analysis using the NCDB, a joint program of the Commission on Cancer (CoC) of the American College of Surgeons and the American Cancer Society, which contains information about patterns of cancer care and treatment outcomes for approximately 70% of the US population from greater than 1500 hospitals 20. The NCDB contains information not included in the Surveillance, Epidemiology, and End Results (SEER) database, including details regarding use of systemic therapy. The data used in the study are derived from a de‐identified NCDB file. As all patient information in the NCDB database is de‐identified, this study was exempt from institutional review board evaluation.

### Patient selection

Inclusion criteria for this study involved newly diagnosed patients aged 65 years or older with nonmetastatic, histologically proven NPC that did not receive surgery and were treated with definitive RT with or without chemotherapy. For the purposes of this study, definitive RT was defined as a radiation dose of ≥6600 cGy, based on national guidelines 2. The tumors were categorized by the histologic types as determined by the World Health Organization (WHO) classification scheme using the ICD‐O‐3 codes. Squamous cell carcinoma (ICD‐O‐3 codes 8070 and 8071) represented the keratinizing squamous cell carcinoma histologic subtype (KSCC, formerly WHO type I); nonkeratinizing carcinomas (ICD‐O‐3 codes 8072 and 8073) represented the differentiated nonkeratinizing carcinoma (NK‐D, formerly WHO type II) histologic subtype; undifferentiated, anaplastic, and lymphoepithelial carcinoma (ICD‐O‐3 codes 8020, 8021, and 8082) formed the undifferentiated nonkeratinizing carcinoma (NK‐U, formerly WHO type III) histologic subtype; carcinoma, not otherwise specified (NOS, ICD‐O‐3 code 8010) formed the NOS histologic subtype. The patients were staged based on the American Joint Committee on Cancer (AJCC) 6th edition classification if diagnosed between 2004 and 2009 or based on the AJCC 7th edition classification if diagnosed between 2010 and 2012.

Exclusion criteria were patients age younger than 65 years old, distant metastatic disease (stage IVc), unknown stage, unknown histology, therapy with palliative intent or treatment with chemotherapy alone, incomplete treatment details, unknown follow‐up time, and lack of information regarding treatment facility, and not receiving all RT at the reporting facility. Patients whose registration was in the form of autopsy/death certificate only and patients with no microscopic confirmation of diagnosis were also excluded. Information collected on each patient broadly included demographic data, clinicopathologic tumor parameters, and treatment facility characteristics. The education variable represents the percentage of the number of adults in the patient's zip code who graduated from high school based on US census data.

### Comorbidity assessment

Comorbid conditions as described by Charlson/Deyo (1992) were mapped from as many as ten reported ICD‐9‐CM secondary diagnosis codes. Charlson Comorbidity Index (CCI), originally proposed by Charlson et al. in 1987 21, and then modified in 1992 22, assigns a score to various chronic medical conditions and uses the sum to predict long‐term mortality. Individual Charlson scores are not provided in the database. Instead, the Charlson scores are summed for each patient and categorized by a value of 0, 1, and 2 or more. CCI was divided into three groups: CCI = 0, which was comprised of patients with no comorbidity or none of the conditions from the score mapping table, CCI = 1, meaning a total Charlson score of 1, and CCI ≥ 2, which represented a total Charlson score of 2 or more. In order to provide further information regarding the rates of comorbidity among the patients in the NCDB with nasopharyngeal cancer, the prevalence of comorbidity was calculated for patients ≥65 years of age excluded from definitive RT and for patients <65 years of age.

### Statistical methods

Patient and tumor characteristics were compared using chi‐squared testing. The outcome measure parameter was OS. Univariate and multivariate analyses as part of Cox proportional hazards modeling were used to identify variables associated OS. Follow‐up was calculated from first day of diagnosis to the day of death or last contact. OS was defined as the time from the date of diagnosis until death from any cause or the end of follow‐up. Actuarial rates were estimated by the Kaplan–Meier method, and survival curves compared using the log‐rank test. Multivariate analysis using a Cox proportional hazards model was used to test the different factors including comorbidity, age, gender, treatment, T and N classification, and histologic type for all patients and also for patients when stratified by histologic subtype. Stata Statistical Package (STATA 12; Stata Corp LP, College Station, TX) was used for all analysis. All tests were two‐sided, and *P* < 0.05 was considered significant.

## Results

**Figure 1:**
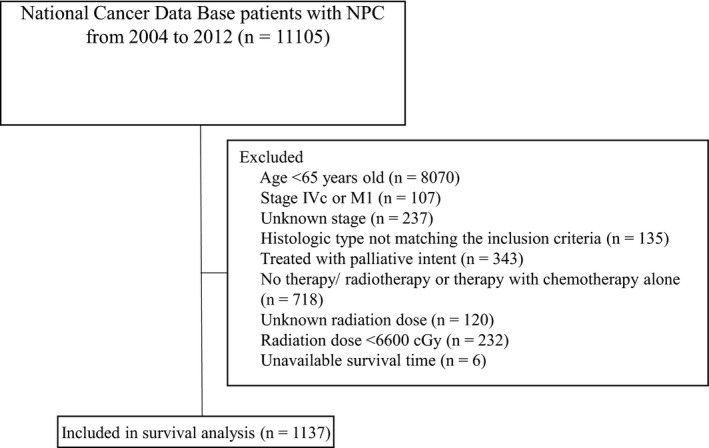
Diagram of analytic cohort for survival analysis.

A total of 1137 patients fulfilled the specified inclusion and exclusion criteria. A complete flow diagram of patient selection is provided in Figure 1. The mean age was 72.2 years (range 65–90; median 71 years), while a total of 152 patients (13.4%) were older than 80 years. The demographic characteristics of this patient cohort are listed in Table 1. The median follow‐up was 61.2 months (range, 2.0–128.9 months).

**Table 1:** Characteristics of elderly patients with nasopharyngeal carcinoma by CCI group

	*N*	Charlson Comorbidity Index			*P*‐value
		0	1	≥2	
Number	1137	882	200	55	
Gender					
Male	787 (69.2)	605 (68.6)	145 (72.5)	37 (67.3)	0.530
Female	350 (30.8)	277 (31.4)	55 (27.5)	18 (32.7)	
Age					
65–69 years	475 (41.8)	377 (42.7)	83 (41.5)	15 (27.3)	0.111
70–74 years	299 (26.3)	235 (26.6)	50 (25.0)	14 (25.5)	
75–79 years	211 (18.6)	157 (17.8)	36 (18.0)	18 (32.7)	
≥80 years	152 (13.3)	113 (12.9)	31 (15.5)	8 (14.5)	
Race					
White	802 (70.5)	625 (70.9)	136 (68.0)	41 (74.5)	<0.001
African American	126 (11.1)	87 (9.9)	38 (19.0)	1 (1.8)	
Other	209 (18.4)	170 (19.3)	26 (13.0)	13 (23.6)	
Histological type					
KSCC	589 (51.8)	444 (50.3)	111 (55.5)	34 (61.8)	0.583
NK‐D	167 (14.7)	132 (15.0)	27 (13.5)	8 (14.5)	
NK‐U	135 (11.9)	108 (12.2)	23 (11.5)	4 (7.3)	
Carcinoma NOS	246 (21.6)	198 (22.4)	39 (19.5)	9 (16.4)	
T stage					
T1	328 (28.8)	259 (29.4)	58 (29.0)	11 (20.0)	0.635
T2	356 (31.3)	272 (30.8)	62 (31.0)	22 (40.0)	
T3	182 (16.1)	136 (15.4)	36 (18.0)	10 (18.2)	
T4	271 (23.8)	215 (24.4)	44 (22.0)	12 (21.8)	
N stage					
N0	377 (33.2)	299 (33.9)	58 (29.0)	20 (36.4)	0.443
N1	336 (29.6)	266 (30.2)	57 (28.5)	13 (23.6)	
N2	321 (28.2)	237 (26.9)	65 (32.5)	19 (34.5)	
N3	103 (9.0)	80 (9.0)	20 (10.0)	3 (5.5)	
Clinical stage					
I	115 (10.1)	94 (10.7)	15 (7.5)	6 (10.9)	0.437
II	305 (26.8)	241 (27.3)	51 (25.5)	13 (2.6)	
III	356 (31.3)	262 (29.7)	73 (36.5)	21 (38.2)	
IVa‐b	361 (31.8)	285 (32.3)	61 (30.5)	15 (27.3)	
Radiotherapy					
Photon	329 (28.9)	260 (29.5)	53 (26.5)	16 (29.1)	0.943
IMRT	674 (59.3)	519 (58.8)	122 (61.0)	33 (60.0)	
3DRT/other	134 (11.8)	103 (11.7)	25 (12.5)	6 (10.9)	
Treatment					
RT	208 (18.3)	161 (18.3)	33 (16.5)	14 (25.5)	0.314
RT + Chemotherapy	929 (81.7)	721 (81.7)	167 (83.5)	41 (74.5)	

CCI, Charlson Comorbidity Index; KSCC, keratinizing squamous cell carcinoma; NK‐D, differentiated nonkeratinizing carcinoma; NK‐U, undifferentiated nonkeratinizing carcinoma; IMRT, intensity‐modulated radiotherapy; 3DRT, three‐dimensional conformal radiotherapy; RT, radiation therapy.

In total, 22.4% (255/1137) of the patients who met the inclusion criteria for the present analysis had comorbidity at the time of diagnosis. No comorbidities (CCI score of 0) were present in 77.6% of the patients, mild comorbidities (CCI = 1) were found in 17.6% of patients, and severe comorbidities (CCI ≥ 2) were present in 4.8% of the patients. Among patients ≥65 years of age not receiving definitive RT, the prevalence of comorbidity was 26.9%. Among patients <65 years of age, the prevalence of comorbidity was 10.2% and 13.2% for patients receiving and not received definitive RT, respectively, and 23.1% of men versus 20.9% of women had were diagnosed at comorbidity (*P* = 0.53). For all histologic subtypes, the distribution according to comorbidity was equal. There was no association between gender, age, histologic type, disease stage, and treatment. However, comorbidity was correlated significantly with race (*P* < 0.001; Table 1). In the group of African American and white patients, at least one comorbidity was present in 31.0% of African Americans and 22.1% of whites (*P* = 0.028); in the group of African American and other race patients, at least one comorbidity was present in 31.0% of African Americans and 18.7% of other race patients (*P* = 0.01). There are no differences between white and other race patients in comorbidity (*P* = 0.284).

**Figure 2:**
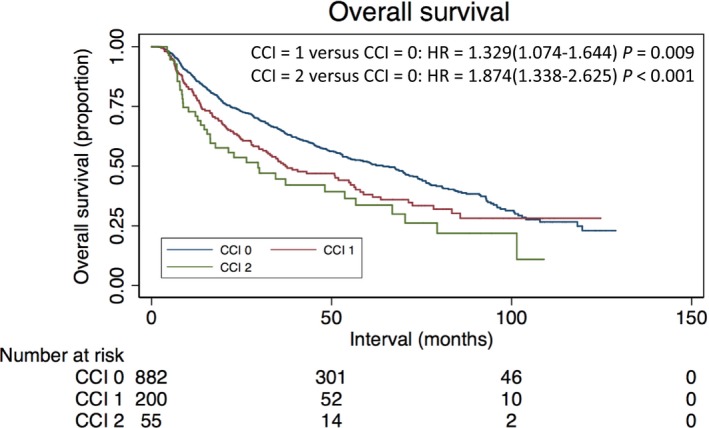
Kaplan–Meier estimates of OS on elderly patients with NPC according to CCI group.

The results of univariate analysis are shown in Table 2. Survival significantly decreased with increasing level of comorbidity, both for patients with a CCI of 1 (hazard ratio [HR]: 1.329; 95% confidence interval [CI]: 1.074–1.644) and for patients with a CCI of ≥2 (HR: 1.874; 95% CI: 1.338–2.635), when compared with patients with a CCI of 0. Age, T classification, N classification, disease stage, and histologic type were also significantly associated with OS in univariate analysis (all *P* < 0.05), with patients with higher T and N classification, advanced disease stage, older age, or keratinizing squamous cell carcinoma displaying poorer OS. Kaplan–Meier curves depicting OS for patient by CCI score are presented in Figure 2. The 5‐year OS rates of patients with CCI score of 0, 1, and ≥2 were 53.1%, 42.2%, and 32.9% (*P* = 0.009).

**Table 2:** Univariable analysis of predictive factors for OS

Factor	No. of patients	5‐year survival (%)	HR	95% CI	*P*‐value
Gender					
Male	787	51.9	1	Reference	
Female	350	46.7	0.998	0.834–1.196	0.986
Age					
65–69 years	475	58.9	1	Reference	
70–74 years	299	54.6	1.049	0.839–1.312	0.674
75–79 years	211	35.9	1.833	1.463–2.297	<0.001
≥80 years	152	31.3	2.142	1.678–2.733	<0.001
Race					
White	802	48.0	1	Reference	
African American	126	54.2	0.926	0.704–1.217	0.581
Other	209	55.5	0.813	0.647–1.023	0.077
Histological type					
KSCC	589	43.3	1	Reference	
NK‐D	167	48.7	0.751	0.581–0.971	0.029
NK‐U	135	68.7	0.483	0.353–0.662	<0.001
Carcinoma NOS	246	57.4	0.717	0.577–0.891	0.003
T stage					
T1–2	684	57.5	1	Reference	
T3–4	453	39.0	1.662	1.403–1.968	<0.001
N stage					
N0–1	713	54.8	1	Reference	
N2–3	424	42.0	1.421	1.198–1.685	<0.001
Clinical stage					
I	115	70.3	1	Reference	
II	305	62.2	1.321	0.899–1.943	0.156
III	356	47.7	1.980	0.365–2.873	<0.001
IVa‐b	361	35.9	2.775	1.923–4.006	<0.001
Radiotherapy					
Photon	329	53.2	1	Reference	
IMRT	674	49.2	1.146	0.945–1.390	0.165
3DRT/other	134	46.0	1.179	0.885–1.570	0.260
Treatment					
RT	208	51.3	1	Reference	
RT + Chemotherapy	929	49.9	0.975	0.788–1.207	0.818
CCI					
CCI = 0	882	53.1	1	Reference	
CCI = 1	200	42.2	1.329	1.074–1.644	0.009
CCI ≥ 2	55	32.9	1.874	1.338–2.625	<0.001

CCI, Charlson Comorbidity Index; KSCC, keratinizing squamous cell carcinoma; NK‐D, differentiated nonkeratinizing carcinoma; NK‐U, undifferentiated nonkeratinizing carcinoma; IMRT, intensity‐modulated radiotherapy; 3DRT, three‐dimensional conformal radiotherapy; RT, radiation therapy.

In multivariate Cox proportional hazard analyses conducted among all patients (Table 3), comorbidity was independently associated with worse OS, with HRs of 1.242 (95% CI: 1.002–1.539) and 1.625 (95% CI: 1.157–2.283) for patients with a CCI score of 1 or ≥2, respectively, when compared with patients with a CCI score of 0. (Table 3) Furthermore, higher T classification (HR: 1.754; 95% CI: 1.479–2.080), nodal disease, (HR: 1.511; 95% CI: 1.271–1.796), age over 75 years old, and histologic type remained independent prognostic factors in the analyses.

**Table 3:** Multivariate analysis of the impact of all variables on overall survival in elderly patients with NPC

	Overall survival			*P*‐value
	Variable	HR	HR (95% CI)	
CCI	CCI = 0	1	Reference	
	CCI = 1	1.242	1.002–1.539	0.048
	CCI ≥ 2	1.625	1.157–2.283	0.005
Age	65–69 years	1	Reference	
	70–74 years	1.104	0.882–1.382	0.389
	75–79 years	1.951	1.552–2.453	<0.001
	≥80 years	2.367	1.849–3.030	<0.001
Histological type	KSCC	1	Reference	
	NK‐D	0.782	0.604–1.012	0.062
	NK‐U	0.497	0.363–0.682	<0.001
	Carcinoma NOS.	0.731	0.588–0.909	0.005
T stage	T1–2	1	Reference	
	T3–4	1.754	1.479–2.080	<0.001
N stage	N0–1	1	Reference	
	N2–3	1.511	1.271–1.796	<0.001

CCI, Charlson Comorbidity Index; KSCC, keratinizing squamous cell carcinoma; NK‐D, differentiated nonkeratinizing carcinoma; NK‐U, undifferentiated nonkeratinizing carcinoma.

Evaluation of multivariate analysis when stratifying by histologic subtype revealed that among KSCC patients, the CCI score appeared to be associated with worse OS, with patients with a CCI score of 1 displaying a strong trend to worse OS (HR: 1.253; 95% CI: 0.962–1.633; *P* = 0.095), and those with a CCI score of 2 displaying a statistically significant association with worse OS (HR: 1.848; 95% CI: 1.199–2.849; *P* = 0.005) when compared to patients without comorbidity. The only other histologic subtype in which the presence of comorbidity was associated with worse OS was the nonkeratinizing undifferentiated carcinoma (NK‐U), in which a CCI score of 1 was associated with worse OS (HR: 2.518; 95% CI: 1.222–5.184; *P* = 0.012) but not a CCI score of 2 (HR: 2.626; 95% CI: 0.594–11.608; *P* = 0.203).

## Discussion

Increased life expectancy has led to an aging population and has increased the proportion patients with cancer who are classified as elderly, particularly in Western countries 3. While elderly patients account for 60% of all new cancer cases, due to strict exclusion criteria, only 36% of patients older than 65 years participate in clinical trials 3, 23. The inadequate representation of elderly patients in clinical trials creates can limit the applicability of these data in this patient population. The present study describes the prevalence of comorbidities in an elderly cohort of patients undergoing definitive radiation for NPC and also shows that the presence of comorbidities independently predicts for worse OS.

In this study of elderly patients with NPC receiving definitive RT, we found that, when using the CCI score as a measure of medical comorbidities, significant comorbidities were present in 22.4% of patients. Previous studies have reported the incidence of comorbidity in patients with NPC to be 40.2–58% 14, 15, 16, 17, 18. Ramakrishnan et al. 18 reported a 44% incidence of comorbidities in 59 patients with NPC in nonepidemic area with cardiovascular and pulmonary diseases most common, though this study used the Adult Comorbidity Evaluation‐27 (ACE‐27) instrument, and not CCI, to measure comorbidities. Guo et al. 17 demonstrated 42.2% of patients with NPC in southern China had comorbidity which also used the ACE‐27; with the most common comorbidity including gastrointestinal disease. While the comorbidity rate in our study of 22.4% was lower than the rates reported in other studies, this may be due to the fact that our study was restricted to a select group of elderly patients who are medically fit to complete definitive RT. However, even when evaluating elderly patients who did not complete definitive RT, the comorbidity rate was 26.9%, which would still be lower than the rate of comorbidities reported in similar studies. Another possible explanation for the observed difference in rate of comorbidities may be the different test used to measure comorbidities. Kallogjeri et al. 24 has demonstrated that the ACE‐27 identified more comorbidities in a larger number of patients in comparison with CCI, as the ACE‐27 method captures additional pancreatic, neuromuscular, psychiatric, a wider range of cardiovascular disease, alcohol and illicit drug use, and obesity information not captured by CCI. Comparisons between general and disease‐specific comorbidity indices have demonstrated that both are equally effective in predicting OS 25, 26.

In our study, statistically significant differences existed in comorbidity scores between patients of different race. Comorbidities were more frequently observed in African American patients (31%) than in white patients (22.1%) or in patients with the race categorized as “other” (18.7%) (*P* = 0.03).The racial disparity in comorbidity may be due to two of the most important comorbidities measured the CCI, diabetes and hypertension, having a higher incidence in African American patients 27, 28, 29.

Our study is the first of its kind to assess the impact of comorbidity on OS in elderly patients with NPC within the United States. We found that the presence of comorbidity was significantly associated with worse OS on both univariate and multivariate analysis. The result of the study is in concordance with previous studies investigating the impact of comorbidity on OS in other cancers such as head and neck cancer, lung, colon, and breast cancer 17, 30, 31, 32, 33, 34. Previous research exploring prognostic factors related to survival in patients with NPC have stated that comorbidity has a strong relationship with these outcomes 14, 16, 17.In a study by Sze et al. 14 from Hong Kong, comorbidity was revealed to be an independent prognostic factor for poor OS in a small study (*n* = 103) of elderly patients with NPC. Guo et al. 17 conducted a study of patients with NPC and found comorbidity to be a significant, independent prognostic factor for OS (HR = 2.027). A study from Taiwan using the CCI also demonstrated that higher CCI score was associated with worse OS 16. However, a single institution review of 59 NPC patients within the United States demonstrated no correlation between comorbid conditions and oncologic outcome 18. The present study is the first within the United States to demonstrate that medical comorbidities independently predicted for worse OS.

There are several possible explanations for why comorbid disease may predict for worse OS in elderly patients with NPC. One possible reason is that the patient may die due to the comorbid condition before dying from the cancer. Another possible reason is that the comorbid disease may weaken the patient, worsening the severity of treatment‐related toxicity. Consequently, the patient may not be able to complete the prescribed treatment or may be more likely to suffer a severe treatment‐related complication. Furthermore, a severe comorbid disease may lead to a delay in diagnosis or may lead the physician to prescribe less intense curative treatment 31.

In addition to comorbid status, another characteristic that should be taken into account when deciding a treatment course for a patient is age [35, 36]. As expected, older age independently predicts for worse OS, with the present data showed that patients with age 75 and older have worse OS on both univariate and multivariate analysis. While each patient is unique, knowledge regarding prognostic factors can aid in making management decisions and both comorbid conditions and age should be taken into account to select the most appropriate treatment decision for each patient.

Although this study was the largest known cohort of elderly NPC patients treated with RT within the United States, our study did have some limitations. The retrospective methodology and potential for selection bias do not substitute for prospective data. Second, the incomplete nature of a large national database and miscoding can never be excluded as a source of bias. The relatively large number of patients included would likely address any such confounding factors. Lastly, the NCDB does not report rates of relapse, cause‐specific survival, treatment‐related morbidity or mortality, or secondary treatments. The database also cannot provide information on individual Charlson scores and the types of comorbidity. Regardless, the information contained herein does offer meaningful conclusions with regard to the impact of comorbidity on OS in elderly NPC patients.

## Conclusions

In this study, we found that, using CCI, comorbidity was present in 22.4% of elderly patients with NPC. When adjusting for other prognostic factors, comorbidity was independently predictive for worse OS. As the numbers of elderly patients with cancer increasing, multidisciplinary therapeutic strategies are required to score and monitor physiological organ reserve and comorbidities in order to optimize the treatment selected for patients. The results of this study lend support that comorbidities should be assessed in prognostic staging of the elderly patients with NPC.

## Conflict of Interest

None. This has never been published before in any form. All the authors of this paper declare that they have no conflict of interests.
